# Breaking the bonds of reinforcement: Effects of trial outcome, rule consistency and rule complexity against exploitable and unexploitable opponents

**DOI:** 10.1371/journal.pone.0262249

**Published:** 2022-02-02

**Authors:** Jukka Sundvall, Benjamin James Dyson

**Affiliations:** 1 University of Helsinki, Helsinki, Finland; 2 University of Alberta, Alberta, Canada; 3 University of Sussex, Sussex, United Kingdom; 4 Ryerson University, Toronto, Canada; University of Warwick, UNITED KINGDOM

## Abstract

In two experiments, we used the simple zero-sum game Rock, Paper and Scissors to study the common reinforcement-based rules of repeating choices after winning (*win-stay*) and shifting from previous choice options after losing (*lose-shift*). Participants played the game against both computer opponents who could not be exploited and computer opponents who could be exploited by making choices that would at times conflict with reinforcement. Against unexploitable opponents, participants achieved an approximation of random behavior, contrary to previous research commonly finding reinforcement biases. Against exploitable opponents, the participants learned to exploit the opponent regardless of whether optimal choices conflicted with reinforcement or not. The data suggest that learning a rule that allows one to exploit was largely determined by the outcome of the previous trial.

## Introduction

When organisms compete for mutually exclusive outcomes, success requires the minimization of losses and the maximization of gains [1]. To achieve these complementary goals, organisms must avoid exploitation (loss minimization) but also be able to exploit their opponents (gain maximization). The sequential play of zero-sum games such as Rock, Paper, Scissors (RPS) or Matching Pennies (MP) are examples of controlled competitive spaces in which the relative success of these goals can be clearly assessed. Within these types of games, the only way to guarantee loss minimization is to behave according to a mixed strategy (MS) [2,3]. A mixed strategy is where no single response option should be played more than another, and the events of the previous round should not influence response selection on the next round. In sum, MS amounts to playing each round randomly. However, the extent to which humans are capable of sequential random decisions has been the source of some debate.

While several early studies seemed to indicate that people have trouble both recognizing and producing randomness (see [4,5] for reviews), critics also identified a number of problems associated with these approaches (see [3,6]). For example, randomness production tasks were often quite artificial, often contained instructional biases (nudging participants towards specific errors in randomness production), and suffered from a lack of incentive and feedback (e.g., no indication to the participant that they are being “sufficiently random”). As a remedy, Rapoport and Budescu suggested two-player zero-sum games would side-step these issues [3]. In a zero-sum game, randomness can become the implicit goal of the game, rather than an explicit task for the participants to “be random.” Zero-sum games are also easy to incentivize and allow for regular feedback (in the form of gains and losses, unlike in many pure randomness production tasks). Rapoport and Budescu found that participants playing a binary-choice zero-sum game against each other were noticeably better at approximating randomness than participants who were simply asked to produce random sequences of game choices without an opponent [3].

Although zero-sum games increase the likelihood of expressing randomness, there remain a number of common predictabilities in behavior based on reinforcement [7]. Actions followed by positive outcomes are more likely to be repeated (*win-stay*) whereas actions followed by negative outcomes are less likely to be repeated (*lose-shift*). That is, game feedback–wins and losses in a zero-sum game–may remove some forms of deviations from randomness, but may give rise to other forms. These associations between outcomes and future behavior are evidenced in both human [8] and animal work [2,9].

We will refer to tendencies to repeat decisions that yielded rewards and to switch away from decisions that did not yield rewards as “reinforcement biases” throughout the paper. We use this term in a similar manner to, e.g., [10,11]. In this tradition of using the term, the word “reinforcement” refers to the specific effect of the immediately previous trial and its outcome on the current decision in a series of decisions. Thus, the term “reinforcement bias” does not cover biases in forms of reinforcement learning where information from more than one previous trial affects decisions. Note that by “bias”, we do not necessarily mean that these decisions are “faulty” or “irrational”. A bias may in fact be rational due to, e.g., evolutionary reasons [12]. Moreover, biases in environments that do not reward or punish predictability *or* unpredictability (e.g., playing against a randomly playing opponent who does not try to exploit the player) do not matter in terms of outcomes. Thus, any way of playing in such an environment could be called “rational”. Our intention in this article is not to make claims about the rationality, or lack thereof, of our participants. Rather, people’s decisions in differing environments may be more or less optimal if an optimal strategy exists: there can be differences in learning. In environments where any strategy leads to the same result, there are no optimal strategies, but we can still examine whether people deviate from randomness in such environments that do not incentivize playing non-randomly.

Reinforcement biases seem quite robust, even in the face of negative feedback. Both Scheibehenne et al. [12] and Wilke et al. [13] found that *win-stay / lose-shift* behavior was common not only in situations where rewards were random, but also when *win-stay / lose-shift* behavior led to a decrease in reward in a simulated slot machine game. Similarly, Achtziger et al. [10] found that in a Bayesian belief updating task, where the reversed *win-shift / lose-stay* strategy was the optimal approach, suboptimal *win-stay / lose-shift* behavior persisted. Thus, it seems that people may adopt a strategy based on reinforcement in the narrow sense (the result of the round immediately before a given round) even when it leads to more frequent losses, with these losses not necessarily being enough to lead to players adopting another strategy.

Although *lose-shift* behavior might seem a simple mirror image of *win-stay* behavior, these mechanisms are under different degrees of control [14–17]. A common finding using three-response zero-sum games (such as Rock, Paper, Scissors) is that *lose-shift* behavior is more frequent than *win-stay* behavior [18,19]. In the following experiments, we explored the tension between the expression of random behavior and behavior guided by reinforcement.

## Experiment 1

In Experiment 1, we explored the joint ability of participants to play randomly when there is no winning strategy, and to play against reinforcement by designing two types of opponent. Our exploitable opponent was designed with a bias towards *shifting* behavior across consecutive trials. In the context of Rock, Paper, Scissors, there are three responses and hence two forms of *shift* available. We chose opponent *downgrading* [20] for Experiment 1, whereby the opponent would *shift* to the response that would have lost to its previous selection (e.g., moving from Rock to Paper). This opponent bias then allows us to clearly define the optimal outcome-response associations for the participant (see Table 1). In order to maximize wins against this exploitable opponent, participants should *win-downgrade* (after winning, *shift* to the response that would have been beaten by your previous response), *lose-upgrade* (after losing, *shift* to the response that would have beaten your previous response), and *draw-stay* (after drawing, repeat your previous response).

**Table 1:** Optimal strategy for trial *n+1* as a function of outcome at trial *n* against exploitable opponents in Experiments 1 and 2.

Experiment	Outcome at trial *n*		
	*Win*	*Lose*	*Draw*
*Experiment 1*	Downgrade	Upgrade	Stay
*Experiment 2*	Upgrade	Stay	Downgrade

We can then assess whether the requirement of optimal responding is in alignment or out of alignment with the standard reinforcement learning principles of *win-stay* and *lose-shift*. Specifically, *win-downgrade* and *draw-stay* behaviors are misaligned with these principles, as participants must change a response following positive outcomes (contra *win-stay*) and maintain a response following negative outcomes (contra *draw-shift*). Only *lose-downgrade* is consistent with changing a response following a negative outcome (*lose-shift*). Additionally, none of the optimal responses are aligned with myopic best reply (i.e., the assumption that the opponent will repeat their last move; see [11]), while the *draw-stay* response is aligned with inertia (repetition bias; see [11]). On the basis that it is difficult to express behaviors other than *win-stay* and *lose-shift* even when such behavior works against the maximization of wins [10,12,13], we predicted that both the proportion of *win-downgrade* and *draw-stay* behavior should be lower than *lose-upgrade* behavior, as a result of the misalignment of *win-downgrade* and *draw-stay* (and alignment of *lose-upgrade*) with reinforcement.

### Method

#### Participants

40 individuals (31 female) from the University of Sussex community participated in the study; mean age was 21.13 years (SD = 4.37) and 39 were right-handed. Sample sizes were based on previous studies from the lab showing reliable lose-shift biases within zero-sum game contexts (e.g., [18], N = 31; [19], Ns = 36; [21], Ns = 40). Participants received course credit or £10 (their choice, unless course enrollment required them to take the credit) for their participation. Informed consent was obtained from all participants before testing, and the experiment was approved by the Sciences Technology Research Ethics Committee (C-REC) at the University of Sussex (ER/JS753/1).

#### Materials

Static pictures of separate white-gloved and blue-gloved hands signaling Rock, Paper and Scissors poses (from [22]) were displayed center screen at approximately 6° x 6°, with participants sat approximately 57 cm away from a 22” Diamond Plus CRT monitor (Mitsubishi, Tokyo, Japan). Stimulus presentation was controlled by Presentation 19 (build 03.31.15) and responses were recorded using a keyboard.

#### Design

Experiment 1 had a 2x2 within-participants design with the factors of opponent (unexploitable, exploitable) and value (low, high; see S1 File). Each participant completed a block of 90 game trials in each of the four conditions (360 trials in total) in a semi-counterbalanced order across participants. The only constraint imposed on the counterbalancing orders was that no two consecutive blocks were allowed to be against the same opponent type; this was to avoid potential ceiling effects against exploitable opponents.

In the unexploitable condition, the opponent drew randomly without replacement from an equal distribution of responses (30 instances of Rock, Paper and Scissors each). Note that this is a slight deviation from true MS, where the draws would be with replacement (this deviation was made to eliminate the possibility of item bias). In the exploitable condition, the computer followed a *downgrade* rule for 70% of the time (63 trials) where the computer’s next response was the one that would have been beaten by the computer’s previous response. For the rest of the time (30%; 27 trials), the computer drew randomly, without replacement, from an equal distribution. This led to the following optimal outcome-strategy contingencies for the participant in the exploitable conditions: *win-downgrade*, *lose-upgrade*, *draw-stay* (see Table 1).

#### Procedure

At the beginning of each block, the experimental program informed participants how much outcomes were worth. Regardless of the opponent condition, participants were informed that their opponent would play in a certain way that would be revealed to them at the end of the experiment. Participants were instructed to try and win as many rounds as possible.

For each trial, the participant was first presented with three pictures of a hand in a white glove representing the three possible choices, presented in the same order as the response buttons used. Opponent and player scores were displayed at the bottom of the screen. Upon pressing a response button, the choices made by the opponent (hand in a blue glove on the left) and the participant (hand in a white glove on the right) were displayed for 1000ms. This was replaced by an outcome screen for 1000ms informing the participant if they had won, lost, or drawn the trial. After a 500ms pause, the scoreboard and trial counter were updated after another 500ms pause, and the next trial began.

For every 9th trial in the block, after the participant had made their choice and before presenting the results, there was a 500ms pause and the program asked the participant to state their confidence of a win or a loss on a 5-point scale. The scale was from 1 for”extremely confident of win” through 3 for”unsure either way” to 5 for”extremely confident of loss”. These items were reverse coded in the final analyses (see S1 File). After another 500ms pause, the trial continued with the outcome reveal. At the end of each block, three short questionnaires were completed (see S1 File) and participants wrote down a short description of how they thought the opponent played. After the final block, a self-report personality inventory was completed, after which participants were thanked for their time and debriefed.

### Results

#### Item selection and outcome at trial n

We analyzed proportions of item selection at trial n for each block with a three-way repeated measures ANOVA with opponent *(unexploitable*, *exploitable)*, value *(low*, *high)* and item choice *(rock*, *paper*, *scissors)* entered as factors (see Table 2). This and all subsequent analyses were conducted using R 4.0.2 [23]. For repeated measures ANOVAs, we used the anova_test function from the rstatix package [24]. In this and all other cases of ANOVAs, degrees of freedom were corrected using Greenhouse-Geisser estimates when Mauchly’s test indicated violations of sphericity. There was no main effect of item choice [F(1.61, 62.90) = 1.27, MSE = .02, *p* = .282, η_p_^2^ = .03] nor an interaction between item choice and value [F(2, 78) = 0.62, MSE < .01, *p* = .538, η_p_^2^ = .01] or item choice and opponent [F(2, 78) = 1.04, MSE < .01, *p* = .358, η_p_^2^ = .02]. There was no three-way interaction [F(1.58, 61.69) = .64, MSE < .01, *p* = 494, η_p_^2^ = .01]. Thus, there was no overall item bias, nor any item biases as a function of experimental condition.

**Table 2:** Item and outcome proportions as a function of opponent and value in Experiments 1 and 2.

*Experiment 1*						
	Unexploitable opponent			Exploitable opponent		
Item choice	Rock	Paper	Scissors	Rock	Paper	Scissors
*Low value*	.345 (.079)	.320 (.084)	.334 (.130)	.348 (.058)	.321 (.057)	.331 (.049)
*High value*	.362 (.069)	.315 (.062)	.323 (.078)	.346 (.058)	.322 (.057)	.333 (.066)
Outcome type	Win	Lose	Draw	Win	Lose	Draw
*Low value*	.335 (.056)	.337 (.053)	.328 (.049)	.499 (.161)	.235 (.104)	.266 (.077)
*High value*	.334 (.046)	.346 (.061)	.320 (.052)	.489 (.161)	.237 (.085)	.274 (.099)
*Experiment 2*						
Item choice	Rock	Paper	Scissors	Rock	Paper	Scissors
*Low value*	.346 (.072)	.330 (.082)	.324 (.093)	.348 (.058)	.321 (.057)	.331 (.049)
*High value*	.371 (.070)	.324 (.075)	.306 (.061)	.364 (.056)	.326 (.058)	.310 (.045)
Outcome type	Win	Lose	Draw	Win	Lose	Draw
*Low value*	.343 (.046)	.337 (.048)	.320 (.050)	.469 (.165)	.257 (.096)	.274 (.109)
*High value*	.332 (.054)	.347 (.064)	.321 (.047)	.478 (.177)	.268 (.100)	.254 (.092)

Note: Standard error in parenthesis.

We also conducted the analysis using a linear mixed model, using the lmerTest package [25], with opponent, value and item choice as fixed effects, and with random intercepts for participants on average and for participants within each main effect and two-way interaction effect. For this analysis, we removed extreme outlier data points, which the ANOVA approach did not allow for (requiring the removal of all of a participant’s data due to one outlier value). We used the rstatix package [24] and its identify_outliers function. Extreme outliers were defined as values that were three times the cell-level interquartile range (Q3 –Q1) above Q3 or below Q1. We detected eight extreme outlier values and removed them from the linear mixed effects analysis. The results differed from the results of the ANOVA only in the main effect of item, which was now significant [F(2, 111.42) = 1.27, *p* = .001, η_p_^2^ = .04]. *Rock* (EMM = 35.3%, 95% CI = [33.8%, 36.7%]) was chosen significantly more often than *paper* (EMM = 32.2%, 95% CI = [30.8%, 33.7%]) or *scissors* (EMM = 31.6%, 95% CI = [30.1%, 33.1%]). Thus, the numerical trend in the data (see Table 2) was significant in this analysis. Conducting the same model without exclusions but with a robust or Bayesian linear mixed effects approach, using the robustlmm and blme packages [26,27], respectively, yielded similar results. Thus, the outlier values seem to have masked a small item bias effect.

We conducted an identical series of analyses for rates of different outcome types *(win*, *lose*, *draw)*. In the three-way repeated measures ANOVA, there was a significant main effect for outcome type [F(1.29, 50.54) = 46.86, MSE = .02, *p* < .001, η_p_^2^ = .54], and a significant interaction between outcome type and opponent [F(1.17, 45.71) = 36.09, MSE = .03, *p* < .001, η_p_^2^ = .48]. There were no significant differences between *wins* and other outcome types in the *unexploitable* condition (33.4%, 34.1% and 32.4%, respectively; Tukey’s HSD; *p* > .05 for both comparisons). In contrast, in the *exploitable* condition, wins were more frequent than losses or draws (49.4%, 23.6% and 27.0%, respectively: Tukey’s HSD; *p* < .05 for both comparisons) confirming that as a group, participants acquired some knowledge of the correct strategy. The main effect of value [F(2, 78) = .24, MSE < .01, *p* = .785, η_p_^2^ < .0], and the three-way interaction [F(1.70, 66.48) = .30, MSE < .01, *p* = .705, η_p_^2^ < .01] were not significant.

We also conducted the outcome analysis using regular (excluding extreme outliers), robust (without exclusions), and Bayesian (without exclusions) linear mixed effects approaches, as we did for item choice data. We detected one extreme outlier. There were no meaningful differences between the results of the different linear mixed effects models, and the results of these models did not differ from the results of the ANOVA. There were also no differences between the results of pairwise comparisons using the full data or the data that excluded extreme outliers.

#### First-order repetition effects

**Fig 1:**
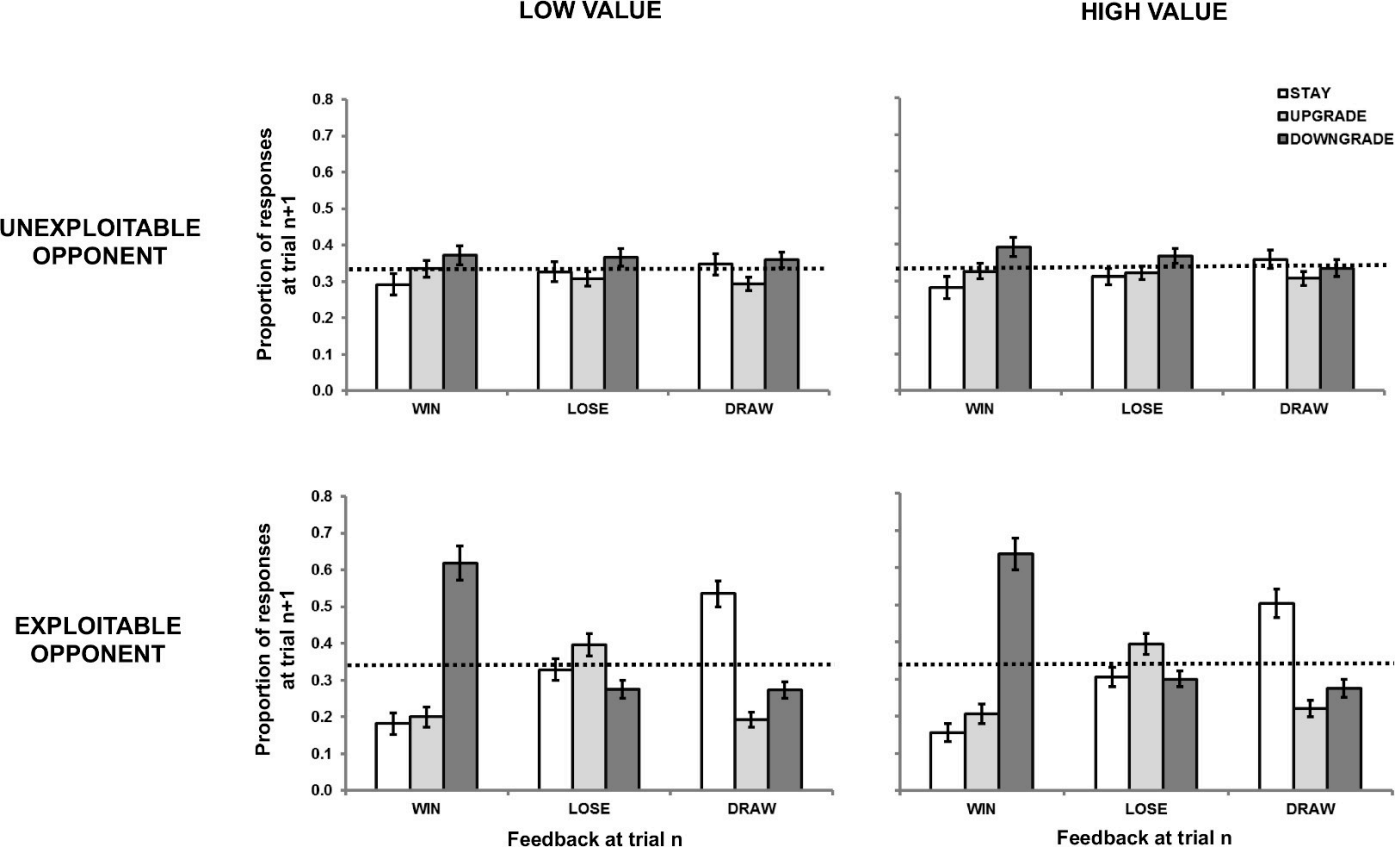
Proportion response data from Experiment 1 in terms of the relationships between the outcome at trial *n* (*win*, *lose*, *draw*) and the strategy selected at trial *n+1* (*stay*, *downgrade*, *upgrade*). Participants competed against four opponents defined by the factors of value (*low*, *high*) and exploitability (*unexploitable*, *exploitable*). Error bars represent standard error.

We conducted a similar series of analyses for first-order repetition effects (i.e., player strategy) as we did for item choice and outcome. We first analyzed proportion data using the last 89 trials in each block (the first trial having no history) with a four-way repeated-measures ANOVA, with opponent *(unexploitable*, *exploitable)*, value *(low*, *high)*, outcome at trial *n (win*, *lose*, *draw)* and player strategy at trial *n+1 (stay*, *upgrade*, *downgrade)* as factors (see Fig 1). The data show no particular pattern of responding in the *unexploitable* condition. In the *exploitable* condition, optimal responses were most likely following *wins*, less likely after *draws*, and least likely after *losses*.

The main effect of strategy at trial *n+1* was significant [F(1.52, 59.24) = 7.73, MSE = .17, *p* = .003, η_p_^2^ = .17]. There was also a significant interaction effect between opponent and player strategy at trial *n+1* [F(2, 78) = 6.77, MSE = .03, *p* = .002, η_p_^2^ = .15], as well as between outcome at trial *n* and strategy at trial *n+1* [F(1.93, 75.09) = 33.98, MSE = .13, *p* < .001, η_p_^2^ = .47]. There was a significant three-way interaction between opponent, outcome at trial *n* and strategy at trial *n+1* [F(2.13, 83.23) = 31.12, MSE = .08, *p* < .001, η_p_^2^ = .44]. The interactions between value and player strategy at trial *n+1* [F(2, 78) = 0.53, MSE = .04, *p* = .589, η_p_^2^ = .01], value, opponent and player strategy at trial *n+1* [F(1.74, 68.03) = 0.45, MSE = .03, *p* = .611, η_p_^2^ = .01] and between all four factors [F(2.95, 114.87) = 0.20, MSE = .03, *p* = .89, η_p_^2^ = .01] were all non-significant, suggesting no effect of the value manipulation on behavior.

Against *unexploitable* opponents, there were no significant differences between the three different strategies at trial *n+1* as a function of outcome at trial n (Tukey’s HSD, *p* > .05 for all comparisons; see top panel of Fig 1). In these regards, the data represent a fair approximation of random behavior at the group level. As an additional test of random behavior, we carried out two-tailed binomial tests for *stay*, *upgrade* and *downgrade* responses after wins, losses and draws for each individual, under the null hypothesis of 33.3% for each test. We classified an individual as having a bias if two conditions were fulfilled. First, the null hypothesis was rejected for a given decision type following a given outcome. Second, the rate of that type of decision following that outcome was *higher* than 33.3% (a rate significantly *lower* than 33.33% would imply a bias toward some other decision type). Moreover, we aggregated *upgrade* and *downgrade* biases into a general *shift* bias: a participant who had either an *upgrade* or *downgrade* bias or both after a given outcome type was classified as having a *shift* bias. There were 9 individuals in the *low value* condition and 11 in the *high value* condition for whom the null hypothesis could not be rejected (p > .05) for any decision type following any outcome type. That is, these participants had no biases in any direction as a function of reinforcement. In general, after any outcome type, the most common pattern of responding was one with no bias either toward or away from reinforcement (see Table 3).

**Table 3:** Numbers of participants with specific biases or no biases in the unexploitable blocks of Experiments 1 (N = 40) and 2 (N = 40).

		Experiment 1			Experiment 2		
		Win	Lose	Draw	Win	Lose	Draw
*Low value*	*Stay bias*	6	8	7	9	10	13
	*Shift bias*	13	11	4	10	11	8
	*No bias*	21	21	29	21	19	19
*High value*	*Stay bias*	5	7	9	9	9	6
	*Shift bias*	16	4	7	15	9	2
	*No bias*	19	29	24	16	22	32

Note: Bias defined by binomial test.

Against *exploitable* opponents, the optimal *downgrade* response following *wins* was more likely than the other two responses, as was the optimal *stay* response following *draws* (Tukey’s HSD, *p* < .05 for each comparison; see bottom panel of Fig 1). However, the proportion of the optimal *upgrade* responses following *losses* did not differ significantly from the other two responses (Tukey’s HSD, *p* > .05 for both comparisons). The proportion of *win-downgrade* responses was not significantly different from *draw-stay* responses (Tukey’s HSD, *p* > .05), but *lose-upgrade* responses were less frequent than *win-downgrade* and *draw-stay* responses (Tukey’s HSD, *p* < 05 for both comparisons). Taken together, the results suggest that the participants’ ability to maintain optimal strategic responding against an *exploitable* opponent was compromised following the experience of *losing* combined with the initiation of a response *aligned* with reinforcement, relative to the experience of *drawing* or *winning* combined with the initiation of a response *misaligned* with reinforcement.

**Fig 2:**
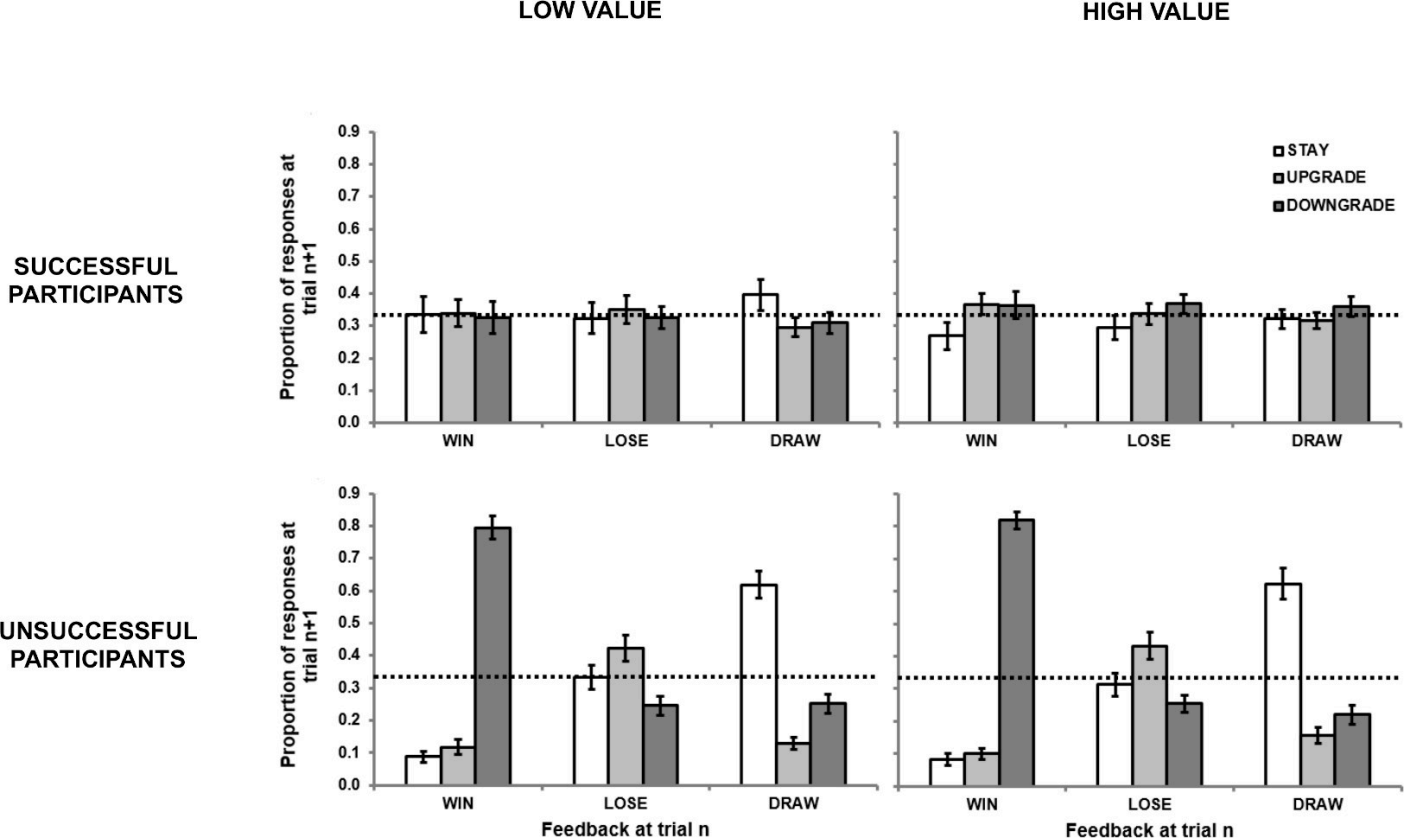
Proportion response data from unsuccessful and successful participants in the *exploitable* condition of Experiment 1 in terms of the relationship between outcome at trial *n* (*win*, *lose*, *draw*) and the strategy selected at trial *n+1* (*stay*, *downgrade*, *upgrade*). Error bars represent standard error.

We further explored the *exploitable* condition data by categorizing participants’ win-rates as successful or unsuccessful based on a one-tailed one-sample proportions test, with 33.3% wins as the null hypothesis, run separately for each participant in each block. Of the 40 participants, 8 failed to reach a win percentage significantly higher than chance on both blocks, and 15 other participants failed on one block out of two (8 in the *low value* and 7 in the *high value* block). For the remaining 17 successful participants, the percentages of optimal responding distributed across the three outcomes was similar to that of the entire sample (see top panel of Fig 2). In contrast, the unsuccessful participants did not show strategic learning following any outcome, and instead behaved similarly to an overall MS trend in both conditions (see bottom panel of Fig 2). Therefore, the observed differences in optimal behavior after different outcomes were not driven by the unsuccessful participants.

We also conducted the first-order repetition effect analysis using regular (with exclusions), robust (without exclusions), and Bayesian (without exclusions) linear mixed effects approaches. In these models, we included the factors in the ANOVA as fixed effects, with random intercepts for participants on average and for participants within each main effect and each two-way and three-way interaction effect. We detected four extreme outliers in the data. There were no meaningful differences between the results of the different linear mixed effects models, and the results of these models did not differ from the results of the ANOVA. There were also no differences between the results of pairwise comparisons using the full data or the data that excluded extreme outliers.

### Discussion

In Experiment 1, behavior against unexploitable opponents was notable in achieving a rough approximation of random behavior in relation to game outcomes at a group level. At the individual level, the predicted pattern of *win-stay*, *lose-shift* and *draw-shift* biases seemed to be as infrequent as non-biased responding. This is quite different from previous studies where participants reliably exhibited a tendency to shift behavior following negative outcomes on the group level during RPS [18,22] or other types of games [12,13]. We did replicate a previously observed, small item bias in favor of choosing rock [8,18,28]. Thus, the results do not fully align with the presumption that humans should find it difficult to produce random behavior [29,30], but we must note that the results are based on measures of very specific kinds of deviations from randomness (item biases and reinforcement biases).

According to Rapoport and Budescu, MS behavior is more likely within the context of game environments where randomness is an optimal, but not explicitly stated, requirement of the task [3]. However, in Rapoport and Budescu’s study, randomness was achieved when a pair of human players played against each other. Human players likely attempt to exploit each other in a dynamic fashion, such that there is no stable strategy that leads to above-chance performance (see [31] for an exploration of a model of how humans play). This is at odds with the use of a computer opponent that fails to produce the same kind of “dynamic coupling” (see [31]) with a human player: the program in Experiment 1 paid no attention to how participants played nor did it attempt to exploit them.

With respect to performance against exploitable opponents, we examined differences in adopting strategies based on whether the strategies aligned with *win-stay / lose-shift* rules [10,13]. In Experiment 1, both win and draw trials required participants’ choices to go against reinforcement in order to be optimal (see Table 1). However, we observed that performance after both wins and draws was better than performance after losses. This suggests that even when the optimal strategy was in alignment with reinforcement for that kind of trial (i.e., *lose-shift*), unambiguously negative outcomes outweighed this potential advantage. Note that since participants on average experienced similar rates of both draws and losses (see Table 2), the participants’ behavior after losses cannot be explained simply due to the low frequency of losses. The possibility remains that poorer performance following losses might be a result of the complexity of the strategy required. In the context of RPS, *shifting* requires the additional step of selecting from one of two responses that are different from the previous trial. This is in contrast to *staying*, which involves the repetition of only a single option. Staying also aligns with decision inertia, i.e., a bias towards simply repeating choices [11]. We addressed this concern in Experiment 2.

## Experiment 2

In Experiment 1, we found that against exploitable opponents with clearly defined counterstrategies, losing led to suboptimal decisions relative to either drawing or winning, even when the specific strategy was consistent with the default *lose-shift* rule. It is further notable that the rate of optimal decisions made after wins was not significantly different from the rate of optimal decisions after draws, even though the proportion of wins (49.4%) exceeded both losses (23.6%) and draws (27.0%). Participants thus had more opportunity to learn the correct exploitable choice following wins relative to draws, but this did not yield higher optimization following wins. Furthermore, participants also had roughly equal opportunity to learn the pattern of outcome-response contingencies following both draws and losses. However, given the difference between the rates of optimization following draws and losses, it would appear losses impact decision-making in ways that draws do not (similar to [32,33]). However, a problem with this interpretation is that the nature of the present outcome might interact with the complexity of the future action required by it. Specifically, *shifting* in a three-response game such as RPS requires at least one more processing step relative to *staying*: not only does one have to decide to switch, one has to decide which response to switch to. Therefore, *lose-upgrade* might be more difficult that *draw-stay* because of the requirement to *upgrade*, and not the actual loss.

To test this idea, we reconfigured the outcome-response pairings in Experiment 2 such that the optimal choice after losses was to *stay*. The other optimal responses were *win-upgrade* and *draw-downgrade* (see Table 1). Thus, the optimal strategy after a draw was now more complex but in line with reinforcement, whereas the optimal strategy after a loss was simpler but misaligned with reinforcement and aligned with decision inertia. Again, none of the optimal responses aligned with myopic best reply. If the reduction in optimal performance following losses in Experiment 1 was due to *stay* responses being easier than specific *shift* responses, then the difference in rates of optimal responding after losses and draws should reverse in Experiment 2.

### Method

40 individuals (28 female) from the University of Sussex community participated in the study; mean age was 22.95 years (SD = 5.53) and 36 were right-handed. Participants received a flat £10 reward for their participation and an average £2 extra for performance. The protocol was approved by the Sciences Technology Research Ethics Committee (C-REC) at the University of Sussex under ER/JS753/5.

The core of Experiment 2 was identical to Experiment 1 in terms of within-participant manipulations of both opponent *(unexploitable*, *exploitable)* and value *(low*, *high)* across four counterbalanced conditions (90 trials each). The *low value* conditions were associated with points only (+1, -1, 0 for wins, losses and draws, respectively), whereas the *high value* conditions were associated with the same points but also converted to money at the end of the experiment at the rate of 10p per point. If the final score summed across both *high value* conditions was zero or negative due to a high number of losses, the participant received the baseline £10 as compensation. *Unexploitable* and *exploitable* opponents were identical to Experiment 1, apart from a reconfiguration of the required outcome-action associations required for successful opponent exploitation: *win-upgrade*, *lose-stay*, and *draw-downgrade* (see Table 1). For analyses of outcome value and player confidence, see S2 File.

### Results

#### Item selection and outcome at trial n

We analyzed item selection and outcome at trial *n* in an identical manner to Experiment 1 (see Table 2). In the three-way repeated measures ANOVA, there was only a significant main effect of item [F(2, 78) = 5.94, MSE = .01, *p* = .004, η_p_^2^ = .13]. Participants were more likely to choose *rock* than *paper* or *scissors* (35.72%, 32.53% and 31.76%, respectively; Tukey’s HSD, *p* < .05 for both comparisons). There was no two-way interaction between item choice and opponent [F(2, 78) = 0.22, MSE = .004, *p* = .804, η_p_^2^ = .01] or item choice and value [F(2, 78) = 2.64, MSE = .01, *p* = .078, η_p_^2^ = .06], and no three-way interaction [F(2, 78) = 0.23, MSE < .01, *p* = .792, η_p_^2^ = .01]. The item bias towards *rock*, not present in Experiment 1, is in line with previous findings [8,18,28].

We also analyzed the item choice data with regular (with exclusions), robust (without exclusions), and Bayesian (without exclusions) linear mixed effects approaches, as in Experiment 1. We detected five extreme outliers in the data. In the linear mixed effects model using the data with exclusions, the interaction effect between item choice and value became significant [F(2, 78) = 2.64, *p* = .039, η_p_^2^ = .02]. The item bias towards *rock*, also observed in Experiment 1, was significant in the *high value* condition only (Tukey’s HSD; p < .05 for all comparisons within the *high value* condition; p > .05 for all comparisons within the *low value* condition). The results of the robust and Bayesian linear mixed effects models were similar. The results of these analyses were otherwise not meaningfully different from the results of the ANOVA.

In terms of outcome at trial *n*, and as in Experiment 1, outcome distributions against the *unexploitable* opponent were roughly uniform (33.75% wins, 34.17% losses and 32.08% draws), while win-rates were above 33.3% against the *exploitable* opponent (47.35% wins, 26.24% draws and 26.42% losses). This was reflected in a significant main effect of outcome [F(1.18, 46.13) = 28.27, MSE = .04, *p* < .001, η_p_^2^ = .42] and a significant interaction between opponent and outcome [F(1.28, 49.85) = 25.58, MSE = .03, *p* < .001, η_p_^2^ = .40]. *Wins* were significantly more frequent than both *losses* and *draws* against the *exploitable* opponent (Tukey’s HSD; *p* < .05 for both comparisons) As in Experiment 1, the results indicate group success in opponent exploitation. The overall win-rate for Experiment 2 was not significantly different from the win-rate observed for Experiment 1 (47.35% versus 49.40%; t[78] = 0.607, p = .546). The interaction between value and outcome [F(2, 78) = 0.58, MSE = .01, *p* = .56, η_p_^2^ = .015] and the three-way interaction [F(1.55, 60.52) = 0.73, MSE = .0, *p* = .454, η_p_^2^ = .02] were not significant.

We detected no extreme outliers in the outcome data. Nevertheless, we also analyzed the outcome data with regular, robust, and Bayesian linear mixed effects approaches, as in Experiment 1. The results of these models did not differ meaningfully from the results of the ANOVA.

#### First-order repetition effects

**Fig 3:**
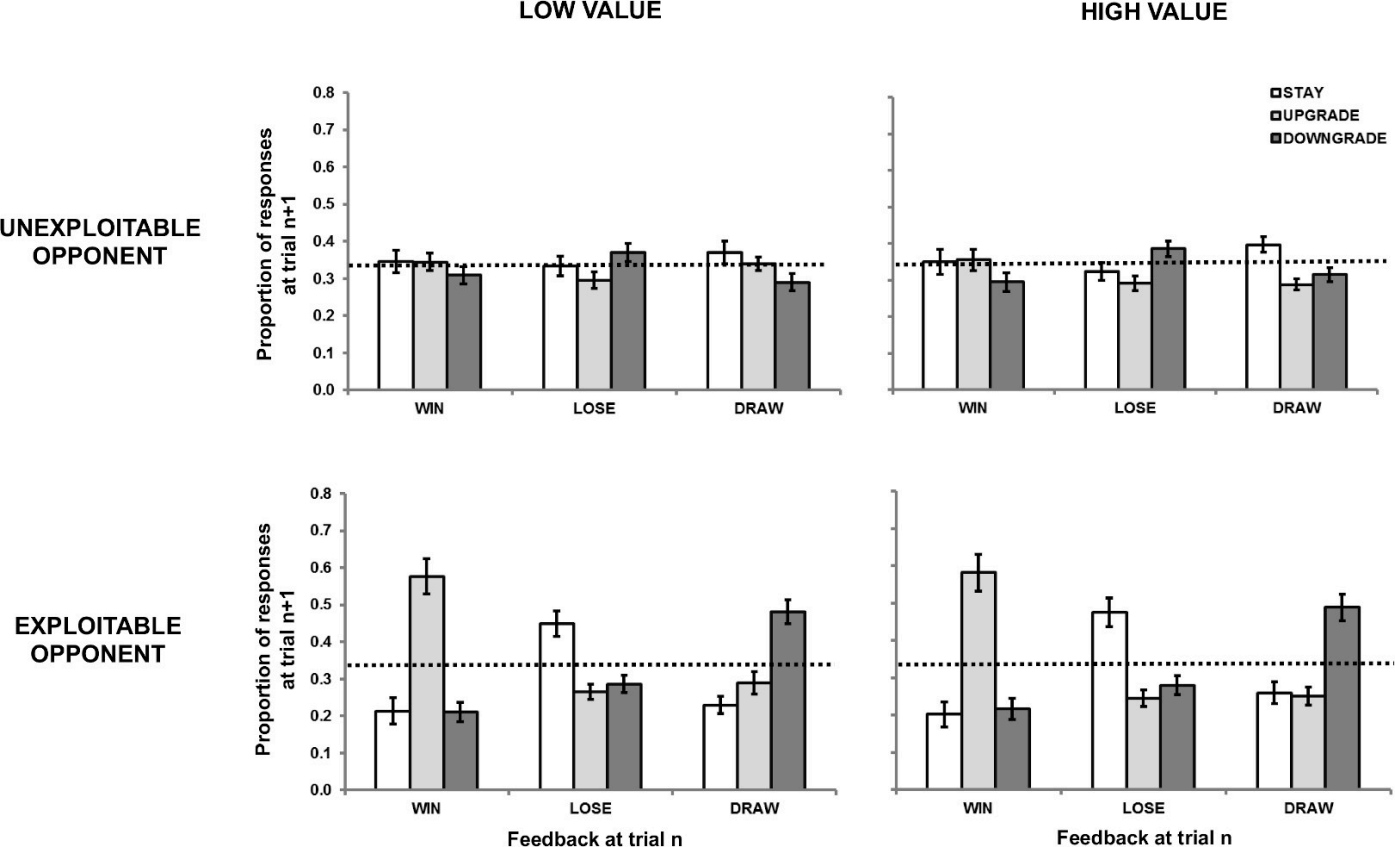
Proportion response data from Experiment 1 in terms of the relationships between the outcome at trial *n* (*win*, *lose*, *draw*) and the strategy selected at trial *n+1* (*stay*, *downgrade*, *upgrade*). Participants competed against four opponents defined by the factors of value (*low*, *high*) and exploitability (*unexploitable*, *exploitable*). Error bars represent standard error.

We analyzed proportion data using the last 89 trials in each block as per Experiment 1 (see Fig 3). As before, there was no discernible pattern of behavior against *unexploitable* opponents. Against *exploitable* opponents, the rank order of outcomes producing optimal responses (more likely to least likely) was *wins*, then *draws*, then *losses*.

The main effect of player strategy at trial *n+1* was not significant [F(1.48, 57.84) = 0.34, MSE = .15, *p* = .646, η_p_^2^ = .01]. However, there were significant interactions between opponent and player strategy at trial *n+1* [F(2, 78) = 9.91, MSE = .03, *p* < .001, η_p_^2^ = .20] and between outcome at trial *n* and player strategy at trial *n+1* [F(2.34, 91.26) = 18.33, MSE = .14, *p* < .001, η_p_^2^ = .32], indicating that player choices of *staying*, *upgrading* and *downgrading* were affected by the outcomes of previous trials as well as opponent exploitability. Further, there was a significant three-way interaction between opponent, outcome at trial *n*, and player strategy at trial *n+1* [F(1.93, 75.26) = 25.14, MSE = .12, *p* < .001, η_p_^2^ = .39], replicating Experiment 1. There was no significant interaction between value and player strategy at trial *n+1* [F(1.67, 65.03) = 0.73, MSE = .04, *p* = .464, η_p_^2^ = .02]. There was also no significant three-way interaction between opponent, value and player strategy at trial *n+1* [F(2, 78) = 0.05, MSE = .03, *p* = .947, η_p_^2^ < .01], no significant three-way interaction between value, outcome and player strategy at trial *n+1* [F(3.34, 130.27) = 1.09, MSE = .02, *p* = .359, η_p_^2^ = .03], and no four-way interaction [F(2.51, 97.90) = 0.25, MSE = .04, *p* = .825, η_p_^2^ < .01].

When playing against the *unexploitable* opponent, there were no significant differences between strategy at trial *n+1* as a function of outcome at trial *n* (Tukey’s HSD; *p* > .05 for all comparisons; see top panel of Fig 3), replicating Experiment 1. This again suggests that participants, on the group level, were able to approximate random responding with regard to previous outcomes and were not generally biased by reinforcement when playing against *unexploitable* opponents. As in Experiment 1, we also carried out binomial tests for each response and outcome type, separately for each individual participant in the unexploitable conditions, under the null hypothesis of 33.3% for each analysis. As in Experiment 1, for a handful of participants—7 individuals in the low value condition and 9 in the high value condition—the null hypothesis could not be rejected (*p* > .05) for any decision types following any outcome type. That is, these participants had no biases in any direction as a function of reinforcement. As in Experiment 1, after any specific outcome type, the most common pattern of responding was not biased either towards or away from reinforcement (see Table 3).

In the *exploitable* condition, the optimal choice of *upgrading* following a *win*, *staying* following a *loss*, and *downgrading* following a *draw* were all significantly more likely than either of the suboptimal choices following each outcome (Tukey’s HSD; *p* < .05 for all comparisons; see bottom panel of Fig 3). However, in Experiment 2, there were no significant differences in rates of optimal decisions between *win*, *lose* and *draw* trials (Tukey’s HSD; *p* > .05 for all comparisons). This was contrary to Experiment 1, where the rate of optimal decisions following *losses* was significantly lower than that following *wins* or *draws*.

**Fig 4:**
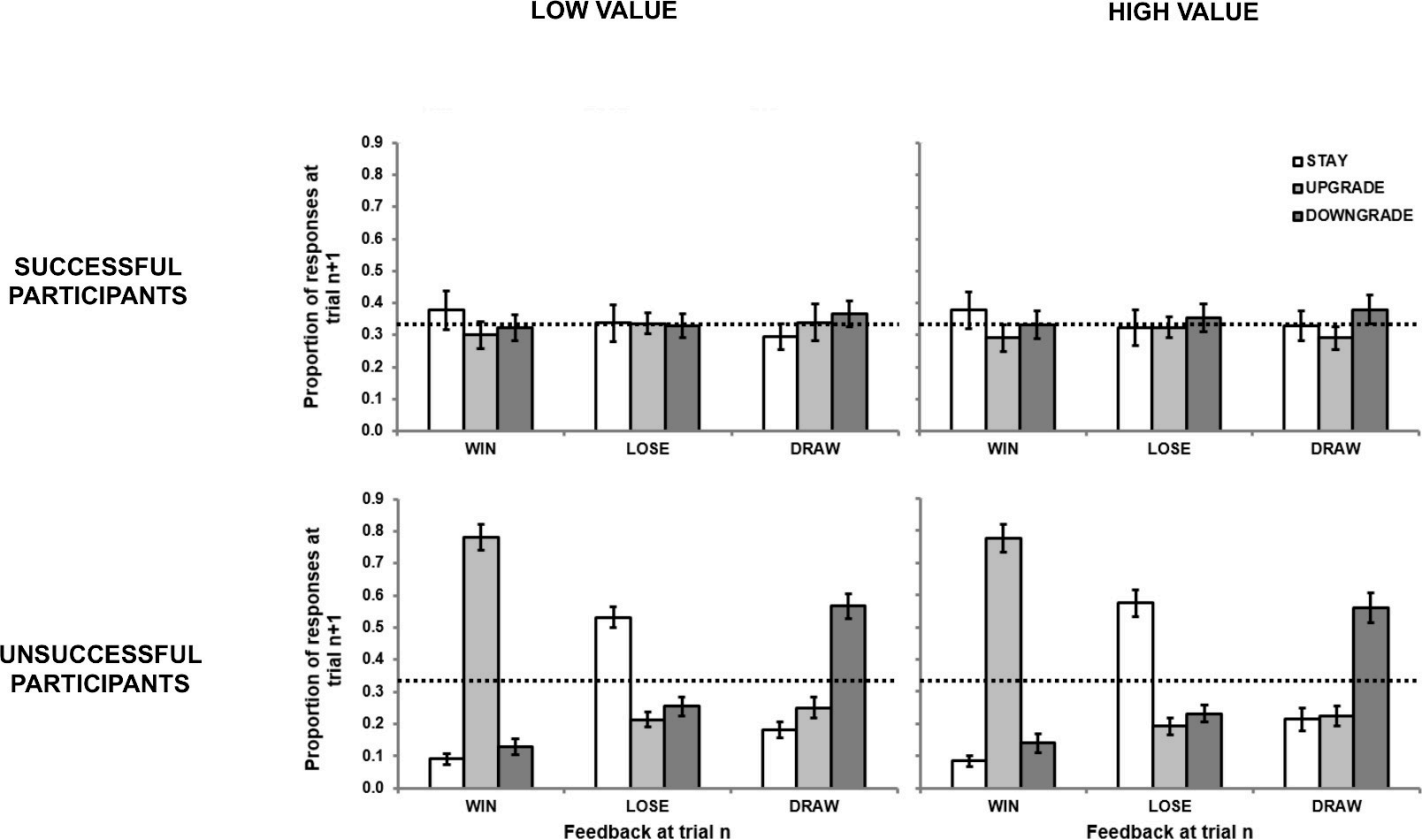
Proportion response data from unsuccessful and successful participants in the *exploitable* condition of Experiment 2 in terms of the relationship between outcome at trial *n* (*win*, *lose*, *draw*) and the strategy selected at trial *n+1* (*stay*, *downgrade*, *upgrade*). Error bars represent standard error.

Similar to Experiment 1, we categorized participants in the *exploitable* blocks as successful or unsuccessful. Out of the 40 participants, 10 failed to reach a win-rate significantly higher than chance on both blocks, with a further 12 failing on one of the blocks (5 for the *high value* block and 7 for the *low value* block). As in Experiment 1, the data of the remaining 18 successful participants was similar to that of the whole sample (see top panel of Fig 4). The unsuccessful participants played essentially randomly, with no strategic learning observable after any outcome (see bottom panel of Fig 4). As in Experiment 1, it seems that the overall results were not skewed by the unsuccessful participants.

There was no significant interaction between value and player strategy at trial *n+1* [F(1.67, 65.03) = 0.73, MSE = .04, *p* = .464, η_p_^2^ = .02]. There was also no significant three-way interaction between opponent, value and player strategy at trial *n+1* [F(2, 78) = 0.05, MSE = .03, *p* = .947, η_p_^2^ < .01], no significant three-way interaction between value, outcome and player strategy at trial *n+1* [F(3.34, 130.27) = 1.09, MSE = .02, *p* = .359, η_p_^2^ = .03], and no four-way interaction [F(2.51, 97.90) = 0.25, MSE = .04, *p* = .825, η_p_^2^ < .01].

We also conducted the first-order repetition effect analysis using regular (with exclusions), robust (without exclusions), and Bayesian (without exclusions) linear mixed effects approaches as in Experiment 1. We detected two extreme outliers in the data. There were no meaningful differences between the results of the different linear mixed effects models, and the results of these models did not differ from the results of the ANOVA. There were also no differences between the results of pairwise comparisons using the full data or the data that excluded extreme outliers.

#### Cross-experiment comparison of optimal choices

To assess the central question of whether relationships between outcome and strategy were equally challenging against exploitable opponents in Experiments 1 and 2, we conducted a Bayesian model comparison of models. The rationale for this analysis was to test whether the difference between the significant effect of outcome on optimal decisions in Experiment 1 was truly different from the non-significant effect in Experiment 2. That is, whether the difference between significant and non-significant was itself significant or not [34].

We included only optimal decisions in the analyses. We first checked the optimal decision data for extreme outliers in each separate condition, i.e., grouping by outcome (win, lose, draw), value (high, low), and experiment (Experiment 1, Experiment 2). We found no extreme outliers, so we conducted the analyses without any exclusions.

Initially, we had conducted a three-way mixed ANOVA on rates of optimal choices, with outcome *(win*, *lose*, *draw)* and value *(low*, *high)* entered as repeated measures factors and experiment entered as the grouping variable (see Figs 1 and 3). Since the most crucial effect in this model, the interaction between experiment and outcome (indicating whether the effect of outcome on performance differed between the experiments) was marginal [F(2, 156) = 2.86, MSE = .11, *p* = .061, η_p_^2^ = .04], we used a Bayesian approach to examine this effect.

We created two alternative linear mixed models, using the lmerTest package [25], with the rate of optimal decisions (i.e., playing the winning strategy) as the dependent variable, outcome *(win*, *lose*, *draw)* and experiment *(Experiment 1*, *Experiment 2)* as fixed effects, and participant ID as a random effect, allowing variability in the intercept but not slopes. The difference between the two alternative models was in whether we included an interaction term: Model 1 included both the main effects of outcome and experiment and their interaction, whereas Model 2 included only the main effects. We did not include the effect of value, as the value manipulation did not seem to affect rates of optimal decisions in either experiment nor the mixed ANOVA comparing the experiments. Thus, by comparing the models we could see whether the data had a better fit with a model that allowed only overall differences between outcomes and experiments, or a model that allowed for a difference between experiments in how outcomes affected decisions.

We used the bayesfactor_models function from the bayestestR package [35] to compare the models. We tested Model 1 (including the interaction between factors) against Model 2 (without the interaction) as the null: the test yielded a Bayes factor of 0.001, indicating substantial evidence in favor of the null. (Vice versa, testing Model 2 against Model 1 as the null yielded a Bayes factor of 818.64, indicating substantial evidence against the null.) Thus, Model 2 was clearly the better model of the two. This model had a significant main effect of outcome [F(2, 398) = 31.52, *p* < .001, η_p_^2^ = .131] and no significant main effect of experiment [F(1,78) = 0.02, *p* = .889, η_p_^2^ = .000]. Neither including the value condition and interactions involving value nor using Bayesian linear mixed effects models in the models to be compared changed this result. Thus, there was no evidence of any overall difference in optimal decision-making between the experiments.

Averaging over the experiments, optimal decisions were the most common following *wins* (EMM = 60.3%, 95% CI = [55.9%, 64.8%]), second most common following *draws* (EMM = 50.2%, 95% CI = [45.8%, 54.7%]), and the least common following *losses* (EMM = 42.8%, 95% CI = [38.4%, 47.3%]). All pairwise comparisons between the outcome conditions were significant (Tukey’s HSD; *p* < .05 for each comparison).

In sum, the result of the cross-experiment comparison suggests that there was no difference between the results of Experiments 1 and 2 in terms of performance. Rather, a model where only the outcome of the previous round affected the rate of optimal responding against an exploitable opponent describes the data best. The results suggest that performance was best after unambiguously positive (*win*) outcomes, followed by ambiguously negative or neutral (*draw*) outcomes, with unambiguously negative (*lose*) outcomes leading to the worst performance. Given the differences between experiments in how the optimal strategies to follow after *losses* and *draws* aligned with reinforcement and/or inertia, the results suggest that such alignment did not affect learning.

### Discussion

Regarding performance against unexploitable opponents, the data from Experiment 2 replicate Experiment 1, with participants behaving in accordance with a fair approximation of MS, at least when it comes to reinforcement-based deviations from randomness. These results are inconsistent with our previous studies in which participants expressed clear *lose-shift* biases against unexploitable opponents [18,22] and other studies using other types of games [12,13]. A potential explanation for the lack of outcome-response biases would be that participants in our previous studies encountered opponents only of an unexploitable nature. Thus, when the design allows for exposure to both unexploitable and exploitable opponents across separate blocks, this may improve the likelihood of random behavior in the unexploitable case (see also [19], Experiment 2). Such a speculative hypothesis would require further testing, perhaps using between-participants designs manipulating the context within which unexploitable opponents are encountered.

## General discussion

First, the present studies challenge the view that humans would always find it difficult to behave randomly [29,30], at least when it comes to reinforcement-based deviations from randomness. Our demonstrations of randomness within simple games are consistent with Rapoport and Budescu’s critiques of empirical studies into subjective randomness [3]. Traditionally, the evaluation of subjective randomness has taken the form of explicit production tasks where the nature of instruction and measurement may bias the participant towards non-random behavior. Rapoport and Budescu argued that it is more likely that randomness is expressed within a dyadic zero-sum game: a task where random behavior is optimal to—but not explicitly specified within—the task. West and Lebiere later provided a plausible model of how mutual attempts at exploiting an opponent’s patterns of play can lead to random choices for both players in the long term [31]. Humans are more likely to exhibit predictable behavior when there is no threat of exploitation [36]. However, participants in our studies produced random-like behavior on the group level without a second human agent and without any attempt from the opponent to exploit the participants (unlike [3,31]). Thus, mutual attempts at exploitation may not be a necessary condition for MS-like behavior in humans. The only overall biases we observed were not reinforcement biases at all, but very modest item biases, which replicated previous findings: participants were slightly more likely to choose rock than other items [8,18,28].

However, we must also note differences between studies in how randomness is measured. For example, Rapoport and Budescu measured several indices of non-random decisions including different lengths of item choice patterns and conditional probabilities on both the group and individual level [3]. In more recent studies on reinforcement biases specifically, simple group averages of conditional probabilities (such as probabilities of *stays* and *shifts* conditional on the outcome of the previous trial) are common [10–12]. Our studies are in this latter, narrower tradition, as we focused on reinforcement biases specific to situations with feedback, such as zero-sum games. While we have described participant behavior as MS-like, it is only in the context of the kinds of deviations from randomness that show up reliably even in game studies (in contrast to pure randomness production tasks). However, we also examined individual-level biases in our samples and found that participants who had no biases either toward or away from reinforcement against unexploitable opponents did not constitute a majority of our samples. Our results do not imply that reinforcement biases such as *win-stay* and *lose-shift* do not exist, but they do question the nomothetic view that such biases are a general property of human cognition. Individuals can avoid biases or even have biases *against* reinforcement, and aggregate statistics may mask some of these patterns (see [36–38]).

Second, the present studies did not find evidence for a reinforcement bias effect in learning optimal strategies against exploitable opponents. Initially, we reasoned that strategies in line with *win-stay / lose-shift* should be learned more easily than strategies that were misaligned with these reinforcement rules [10–13]. Against this idea, performance in Experiment 1 was better in the two cases in which the optimal response was not aligned with reinforcement (i.e., *win-shift*, *draw-stay*), relative to the one case where there was alignment (i.e., *lose-shift*). Our interim conclusion was that the experience of unambiguously negative outcomes compromises performance in ways that other outcomes do not [39]. One charge against this claim was that *shift* rules might simply have been harder to implement than *stay* rules in games with more than two choice options. To initiate a *stay* decision, the player must be able to represent the previous response. To initiate a *shift* decision, the player must not only reject their representation of the previous response, but must additionally draw upon an inventory of alternative actions and select appropriately from them. As the number of possible responses within the game increases, so too does the size of the inventory of alternative actions and the complexity of the selection process. Alternatively, *stay* rules may have been easier to learn due to alignment with inertia, regardless of the number of *shift* options.

In Experiment 2, we addressed these concerns with an exploitable opponent that required to implement a *lose-stay* rule in order to play optimally. This rule was misaligned with reinforcement but reduced the complexity of the decision and aligned the decision with inertia. Contrary to Experiment 1 and our expectations, we observed that the rate of optimal *lose-stay* decisions in Experiment 2 was not significantly different from the rate of optimal *win-shift* and *draw-shift* decisions required against exploitable opponents.

However, when comparing between the two experiments, there were no significant differences in the rates of optimal choices after any given outcome type. The results of the cross-experiment comparison suggest that alignment with reinforcement and/or inertia either played no role or was swamped by the effect of specific outcomes. Note that since none of the optimal strategies in Experiments 1 and 2 were aligned with myopic best reply, we cannot be certain whether the results would have been the same if some of the optimal *shift* responses had aligned with it. However, since this was true for all optimal strategies, we can be certain that learning different outcome-strategy pairings was not affected differently by alignment with myopic best reply.

Optimal responses were the least likely following unambiguously negative (lose) outcomes, followed by ambiguous outcomes (draw), and the most likely following unambiguously positive (win) outcomes (across Experiments 1 and 2; 42.8%, 50.2% and 60.3%, respectively). Better performance after wins may simply reflect the fact that after participants had learned some approximation of the correct strategy, they would naturally win more. Due to this, they would also have more opportunities to solidify their understanding of the correct move to make after a win than after a draw or a loss. In sum, participants had more chances to learn what to do after winning. However, performance after losses was poorer than after draws, suggesting that losses inhibited learning–the fact that losses were as frequent as draws means that participants had equal chances to learn. Despite losses and draws both representing a failure to reach a goal, losses appear to impact on decision-making in ways that draws do not [26,27]. Given that the participants performed best after wins and worst after losses, regardless of alignment with reinforcement, the strongest predictor of optimal responding against exploitable opponents was the valence of the previous trial.

## Conclusion

We evaluated the ability of participants to avoid the reinforcement heuristic (*win-stay*, *lose-shift*) in the game of Rock, Paper and Scissors in two ways: in their ability to play randomly when there is no way to exploit their opponent, and in their ability to learn decision rules that did not align with reinforcement. Against unexploitable opponents, participants exhibited no evidence of any reinforcement biases on average, and instead *stayed* and *shifted* randomly after each outcome type. Against exploitable opponents, participants were less successful after losses than draws, regardless of alignment with reinforcement, and despite equal learning opportunities. The data suggest that humans are able to break the bonds of seemingly robust reinforcement biases, and that the valence of the feedback itself can affect learning more than reinforcement.

The results do not disprove the importance of reinforcement, but they do contradict the assumption that reinforcement is always a significant factor in deviations from randomness or from optimal strategies that do not align with reinforcement. Future research needs to unpack a number of potentially interacting factors regarding the differences between putative “failure” states, including the over-weighting of losses [39], initial neural responses to losses relative to draws [19], and the generation and regulation of negative affect following failure [40]. In addition to this, the interaction between different outcome types and the alignment of optimal choices after specific outcomes with reinforcement, inertia and myopic best reply is a good candidate for future research. Finally, future studies should examine, with between-participants designs, whether exposure to randomly and non-randomly playing opponents, as opposed to only randomly playing opponents, affects the likelihood of reinforcement biases.

## Supporting information

S1 File Supplementary analyses of Experiment 1. (DOC)

S2 File Supplementary analyses of Experiment 2. (DOC)

S3 File Data codebook for S1–S3 Datasets. (DOC)

S1 Dataset Main experimental data, including block-specific questionnaires, from Experiment 1. (CSV)

S2 Dataset HEXACO questionnaire data from Experiment 1. (CSV)

S3 Dataset Experimental data, including block-specific questionnaires, from Experiment 2. (CSV)
