# Supplementary material for: Breaking the bonds of reinforcement: Effects of trial outcome, rule consistency and rule complexity against exploitable and unexploitable opponents
Source: PLoS One. 2022 Feb 2;17(2):e0262249. doi: 10.1371/journal.pone.0262249 (PMC8809577; doi:10.1371/journal.pone.0262249)
Supplement: S1 File — (DOC) [file pone.0262249.s001.doc]

In Experiment 1, we were also interested in the degree to which outcome value impacted on the expression of optimal or sub-optimal responding. In terms of outcome value, we applied a non-financial value manipulation, similar to [1]. Against both opponent types, participants competed two blocks where penalties for losses and rewards for wins were equal to each other but both were either of a low value (-1 versus +1) or of a high value (-3 versus +3; see also [2] for a similar manipulation). Based on [1], we expected the *low-value*, *unexploitable* condition to generate an approximation of MS behaviour following wins (i.e., no bias towards *win-stay* behaviour) but *shift* behaviour following losses and also *shift* behaviour following draws (draws are considered another category of negative outcome; see [3]). Furthermore, if the higher value of outcomes *per se* lead individuals towards more predictable behaviour against *unexploitable* opponents, during the *high-value* condition we should observe both *shift* biases following losses and draws and *stay* biases following wins (see also [4]). Finally, the *high-value,* *exploitable* condition should yield overall lower proportions of optimal outcome-response associations than the *low-value,* *exploitable* condition for decisions where the optimal strategy is counter to reinforcement [4].

An additional feature in Experiment 1 was the examination of the relationship between meta-cognition and decision-making quality: in a strategic environment we must know when our losses were due to lack of knowledge and know to do better; in a random environment we must know when our wins were due to luck and know not to make too much of it. However, as a result of over-confidence biases [5], we may perceive control in environments outside of our sphere of influence [6] and take ownership of outcomes over which we have had little or no say [7]. It seems plausible that a valid sense of control might be present in cases where participants play against *exploitable* opponents but an invalid sense of control might also be present when participants play against *unexploitable* opponents. Consequently, we included confidence measures to gauge participants' certainty of wins during performance, as explicit representations of confidence play an integral role in value-based choice processing (e.g., [8-9]). In a rare case of the use of on-line confidence measures within competitive game contexts, [10] showed that confidence ratings increased with time during trials against *exploitable* opponents. We similarly predicted that participants' confidence would correlate with their success in the game in general, but especially during encounters with *exploitable* opponents.

Method

Value Manipulation

In the *low-value* condition, participants gained 1 point for a win and lost 1 point for a loss. In the *high-value* condition, participants gained 3 points for a win and lost 3 points for a loss. In both conditions, a draw yielded 0.

*Confidence Measures*

For every 9th trial in the block, after the participant had made their choice and before presenting the results, there was a 500 ms pause and the program asked the participant to state their confidence of a win or a loss on a 5-point scale. The scale was from 1 for "extremely confident of win" through 3 for "unsure either way" to 5 for "extremely confident of loss". These items were reverse coded in the final analyses, so that a higher number indicates higher confidence in winning. After another 500 ms pause, the trial continued with the outcome reveal.

Questionnaires

Three short questionnaires were administered following the completion of each RPS block to assess participants' engagement with the game, the degree of anthropomorphism assigned to the computer opponent, and co-presence felt between the player and opponent. These questionnaires were used to maintain similarity with previous studies [1, 11]. First, engagement per block was measured using a modified Game Engagement Questionnaire [12]. The items were changed from present to past tense (e.g. “I lose track of time” was modified as “I lost track of time”) and measured on a 5-point Likert scale. Sixteen of the original nineteen items were used: the items excluded from the modified version were 'I played longer than I meant to', ‘I felt like I just couldn’t stop playing’ and ‘I got really into the game’. As the number of game rounds was fixed, items relating to stopping playing did not fit; the last item was removed due to overlap with the other questionnaires. Second, self-reported co-presence and others' co-presence was measured using two modified items from scales by [13] along with five items added by [1]. Third, the degree of anthropomorphism attributed to the opponent was assessed on the basis of [14], where five anthropomorphic states (mind of its own, intentions, free will, consciousness, experienced emotion) and three non-anthropomorphic states (attractive, efficient, strong) were measured on an 11-point Likert scale (see Appendix for list of items). Finally, to explore possible personality effects, the 60-item HEXACO self-report questionnaire [15] was administered at the end of the experiment, yielding the six personality factors of honesty/humility, emotionality, extraversion, agreeableness, conscientiousness and openness.

Results

*Confidence ratings*

Mean confidence rates for each condition were analysed using a two-way repeated measures ANOVA with opponent *(unexploitable, exploitable)* and value *(high, low)* factors. There was a significant main effect of opponent [F(1,39) = 14.93, MSE = .57, *p* < .001, ƞ_p_^2^= .27], no main effect of value [F(1, 39) = 0.13, MSE = .16, *p* = .726, ƞ_p_^2^ < .01] and no two-way interaction [F(1, 39) = 0.46, MSE = .18, *p* = .501, ƞ_p_^2^ = .01]. As a group, participants were more confident of a win against *exploitable* relative to *unexploitable* opponents (see Supplementary Table A1), although this interpretation should be tempered by the observation that average ratings were around 3 (representing "unsure either way").

We also examined the relationship between confidence ratings and individual win rates by calculating correlation coefficients based on 10 data points for each player in each block: each data point consisted of the reported confidence every 9 trials and the average win rate for those preceding 9 trials. Six participants had to be excluded due to having no variance in their reported confidence on one or more blocks. For the remaining thirty-four participants, an average correlation measure for each condition was calculated by averaging Fisher-transformed individual correlation scores, with the averages inverse Fisher-transformed to produce a coefficient. This yielded average correlations of 0.16 *(low value, unexploitable)*, 0.23 *(high value, unexploitable),* 0.33 (*low value, exploitable),* and 0.40 *(high value, exploitable*). All correlations were significantly different from 0 (*p* < .05). We then examined potential differences in the strength of this association with a two-way repeated measures ANOVA with the z-transformed individual correlation coefficients as the dependent value, and opponent *(unexploitable, exploitable)* and value *(low, high)* entered as factors. There was a significant main effect of opponent [F(1,33) = 4.51, MSE = .27, *p* = .041, ƞ_p_^2^ = .12], no significant main effect of value [F(1,33) = 1.91, MSE = .10, *p* = .174, ƞ_p_^2^ = .06], and no significant interaction [F(1,33) < 0.001, MSE = .192, *p* = .993, ƞ_p_^2^ < .01]. Participants had higher z-scores in the *exploitable* (M = .38, SE = .08) compared to the *unexploitable* (M = .20, SE = .05) conditions. The results suggest that confidence ratings, on average, more accurately tracked win-rates when playing against an *exploitable* opponent, but note that the final condition-level correlations were all relatively low (all below .4).

*Questionnaires*

The end-of-block questionnaire data (game engagement, co-presence, anthropomorphism) were entered into separate two-way ANOVAs, with the factors of opponent (unexploitable, exploitable) and value (low, high). Each questionnaire construct was analysed in a separate analysis (see Supplementary Table A2). One participant was removed for analyses due to a failure to complete all questionnaires.

Regarding game engagement, there were no significant main effects for opponent or value, nor a significant interaction effect (all Fs < 1). Regarding co-presence, there was a significant main effect of opponent [F(1,38) = 6.514, MSE = .440, *p* = .015, ƞ_p_^2^ = .146] but no main effect of value or significant interaction (F's < 1). Felt co-presence was higher in the unexploitable condition (M = 2.875, SE = .158) than in the exploitable condition (M = 2.604, SE = .147). Similarly for anthropomorphism, there was a significant main effect of opponent [F(1,38) = 8.167, MSE = 2.515, *p* = .007, ƞ_p_^2^ = .177] and no significant main effect of value or significant interaction (F's < 1). The perceived anthropomorphism of the opponent was higher in unexploitable conditions (M = 3.838, SE = .437) relative to exploitable conditions (M = 3.113, SE = .410).

As an exploratory analysis, we entered the six HEXACO factor scores for Honesty/Humility (M = 3.349, SE = 0.083), Emotionality (M = 3.423, SE = 0.120), Extraversion (M = 3.251, SE = 0.103), Agreeableness (M = 3.154, SE = 0.088), Conscientiousness (M = 3.385, SE = 0.095) and Openness (M = 3.397, SE = 0.087) as covariates into a multiple regression, with average win rate during both low and high value exploitable conditions as the predictor variable. The overall model fails to yield significance [F(6,32) = 0.303, p = .930].

| *Supplementary Table A1.* Confidence measures and correlations with win-rate as a function of opponent and value in Experiment 1 (nominal means) | | | |
| --- | --- | --- | --- |
| On-line confidence measure (range: 1 – 5) | | | |
| *Unexploitable opponent* | | *Exploitable opponent* | |
| *Low value* | *High value* | *Low value* | *High value* |
| 2.880 (.092) | 2.858 (.079) | 3.300 (.112) | 3.368 (.119) |
| Confidence measure / win-rate correlations (mean Fisher transformed z values) | | | |
| *Unexploitable opponent* | | *Exploitable opponent* | |
| *Low value* | *High value* | *Low value* | *High value* |
| .158 (.069) | .232 (.061) | .346 (.091) | .421 (.096) |
| Note: Standard error in parentheses. | | | |

| Supplementary Table A2. Results from the game engagement (range = 1 – 5), felt co-presence (range = 1 – 5) and perceived anthropomorphism (range = 1 – 11) questionnaires administered at the end of each condition in Experiment 1 (nominal means) | | | | |
| --- | --- | --- | --- | --- |
|  | Unexploitable Opponent | | Exploitable Opponent | |
|  | *Low value* | *High value* | *Low value* | *High value* |
| Game Engagement | 2.484 (.096) | 2.532 (.093) | 2.487 (.095) | 2.487 (.093) |
| Felt co-presence | 2.883 (.143) | 2.868 (.120) | 2.564 (.117) | 2.645 (.120) |
| Perceived Anthropomorphism | 3.938 (.365) | 3.738 (.327) | 3.1495 (.330) | 3.077 (.316) |
| Note: Standard error in parentheses. | | | | |

References

1. Forder, L., & Dyson, B. J. (2016). Behavioural and neural modulation of win-stay but not lose-shift strategies as a function of outcome value in Rock, Paper , Scissors. Scientific Reports, 6, 33809. <http://doi.org/10.1038/srep33809>

2. Hochman, G. & Yechiam, E. (2011). Loss aversion in the eye and in the heart: The autonomic nervous system’s responses to losses. *Journal of Behavioral Decision Making*, *24*, 140-156.

3. Holroyd, C. B., Hajcak, G., & Larsen, J. T. (2006). The good, the bad and the neutral: Electrophysiological responses to feedback stimuli. *Brain Research*, *1105*(1), 93–101. <http://doi.org/10.1016/j.brainres.2005.12.015>

4. Achtziger, A., Alós-Ferrer, C., Hügelschäfer, S., & Steinhauser, M. (2015). Higher incentives can impair performance: Neural evidence on reinforcement and rationality. *Social Cognitive and Affective Neuroscience*, *10*(11), 1477–1483. <http://doi.org/10.1093/scan/nsv036>

5. Robins, R. W., & Beer, J. S. (2001). Positive illusions about the self: Short-term benefits and long-term costs. *Journal of Personality and Social Psychology*, *80*(2), 340–352. <http://doi.org/10.1037/0022-3514.80.2.340>

6. Langer, E. J., & Roth, J. (1975). Heads I win, tails it’s chance: The illusion of control as a function of the sequence of outcomes in a purely chance task. *Journal of Personality and Social Psychology*, *32*(6), 951–955. <http://doi.org/10.1037/0022-3514.32.6.951>

7. Thompson, S. C., Armstrong, W., & Thomas, C. (1998). Illusions of control, underestimations, and accuracy: A control heuristic explanation. *Psychological Bulletin*, *123*(2), 143–161. <http://doi.org/10.1037/0033-2909.123.2.143>

8. De Martino, B., Fleming, S. M., Garrett, N., & Dolan, R. J. (2013). Confidence in value-based choice. *Nature Neuroscience*, *16*(1), 105–110. <http://doi.org/10.1038/nn.3279>

9. Folke, T., Jacobsen, C., Fleming, S. M., & De Martino, B. (2016). Explicit representation of confidence informs future value-based decisions. *Nature Human Behaviour*, *1*, 1–8. <http://doi.org/10.1038/s41562-016-0002>

10. Stöttinger, E., Filipowicz, A., Danckert, J., & Anderson, B. (2014). The effects of prior learned strategies on updating an opponent’s strategy in the rock, paper, scissors game. *Cognitive Science*, *38*, 1482–1492. <http://doi.org/10.1111/cogs.12115>

11. Dyson, B. J., Wilbiks, J. M., Sandhu, R., Papanicolaou, G., & Lintag, J. (2016). Negative outcomes evoke cyclic irrational decisions in Rock, Paper, Scissors. Scientific Reports, (6), 1-7. <http://doi.org/10.1038/srep20479>

12. Brockmeyer, J. H., Fox, C. M., Curtiss, K. A., Mcbroom, E., Burkhart, K. M., & Pidruzny, J. N. (2009). The development of the Game Engagement Questionnaire: A measure of engagement in video game-playing. Journal of Experimental Social Psychology, 45(4), 624-634. <http://doi.org/10.1016/jjesp.2009.02.016>

13. Nowak, K. L., & Biocca, F. (2003). The Effect of the Agency and Anthropomorphism on Users' Sense of Telepresence, Copresence, and Social Presence in Virtual Environments. Presence: Teleoperators and Virtual Environments, 12(5), 481­494. <http://doi.org/10.1162/105474603322761289>

14. Epley, N., Akalis, S., Waytz, A., & Cacioppo, J. T. (2008). Creating Social Connection Through Inferential Reproduction. Psychological Science, 19(2), 114-120. <http://doi.org/10.1111/j.1467-9280.2008.02056.x>

15. Ashton, M. C., & Lee, K. (2009). The HEXACO-60: A short measure of the major dimensions of personality. Journal of Personality Assessment, 91(4), 340-5. <http://doi.org/10.1080/00223890902935878>

**Appendix**

**Anthropomorphism questionnaire items (from Epley, Akalis, Waytz, & Cacioppo, 2008).**

“My opponent had a mind of its own”

“My opponent had intentions”

“My opponent had free will”

“My opponent had consciousness”

“My opponent experienced emotions”

“My opponent was attractive”

“My opponent was efficient”

“My opponent was strong”

**Game Engagement Questionnaire (GEQ) items (from Brockmyer et al., 2009).**

“I lost track of time”

“Things seemed to happen automatically”

“I felt different”

“I felt scared”

“The game felt real”

“If someone would have talked to me, I wouldn’t have heard them”

“I got wound up”

“Time seemed to kind of stand still or stop”

“I felt spaced out”

“I wouldn’t have answered if someone talked to me”

“I couldn’t tell that I was getting tired”

“Playing seemed automatic”

“My thoughts went fast”

“I lost track of where I am”

“I played without thinking about how to play”

“Playing made me feel calm”

**Modified co-presence questionnaire items (from Nowak & Biocca, 2003 and Forder & Dyson, 2016).**

From Nowak & Biocca (2003):

“My opponent was intensely involved in our game”

“My opponent seemed to find our game stimulating”

From Forder and Dyson (2016):

“I felt as though my opponent had a strategy that was based on the moves I was making”

“I felt as though my opponent had a strategy that was based on the moves it was making”

“My opponent exhibited a human-like strategy”

“I felt like my opponent was somehow cheating”

“I found this game of RPS rewarding to play”
