# Supplementary material for: Breaking the bonds of reinforcement: Effects of trial outcome, rule consistency and rule complexity against exploitable and unexploitable opponents
Source: PLoS One. 2022 Feb 2;17(2):e0262249. doi: 10.1371/journal.pone.0262249 (PMC8809577; doi:10.1371/journal.pone.0262249)
Supplement: S2 File — (DOC) [file pone.0262249.s002.doc]

In an attempt to make the difference between outcome values stronger in Experiment 2, we compared monetary (*high-value*) and non-monetary (*low-value*) conditions [1]. Monetary reward is assumed to have a relatively uncontroversial effect upon performance and motivation [2] (although see [3] for an alternative position). Specifically, financial incentives have been show to improve performance on simple cognitive tasks such as the Attentional Network Test (ANT) and the Newcastle Spatial Memory Test (NSMT) [4], increase hit rate in a reaction time task [5], and create shifts towards equilibrium strategies and optimal choices [6]. However, based on [7], when the optimal strategy is in conflict with reinforcement, *high-value* contexts can lead to poorer performance through a greater reliance on reinforcement. Given the design of the optimal strategy in Experiment 2, we expected the high value condition to lead to less optimal choices following wins and losses but not draws. This was because *win-shift* and *lose-stay* responses were inconsistent with reinforcement learning principles, but *draw-shift* responses were consistent with them. Moreover, if a financial incentive increases the likelihood of following reinforcement learning rules, we would also expect more *win-stay* and *lose-shift* behaviour against the *unexploitable* opponent.

Finally, we sought to further explain some of the variation in success rates in Experiment 1. We added a working memory (WM) task to Experiment 2 (Operation Span task; after [8-9]), hypothesizing that successful performance against *exploitable* opponents should be related to WM capacity, given the rule-based structure associated with win maximization. We also predicted that lower WM capacity would predict a better approximation of mixed-strategy against *unexploitable* opponents, as temporarily reducing WM capacity leads to better performance when attempting to produce random sequences [10]. As [10] also found evidence that participants with high levels of executive control were better at producing random sequences, we also added a flanker task [11] to measure executive control. As these data were uninstructive, we include the results in Supplementary Materials B.

Method

Value Manipulation

The *low value* conditions were associated with points only (+1, -1, 0 for wins, losses and draws, respectively), whereas the *high value* conditions were associated with the same points but also converted to money at the end of the experiment at the rate of 10p per point. If the final score summed across both high value conditions was negative due to a high number of losses, the participant received the baseline £10 as compensation.

*Confidence Measures*

As per Experiment 1

Questionnaires

A short questionnaire was administered following the completion of each condition to assess participants' interpretation of the opponent's behaviour and their own success in the game. Participants first indicated their attribution of their game results in the block to luck or skill by clicking on a point on a slider ranging from "100% luck" to "100% skill". The middle-point of this slider was scored as 0, with negative scores indicating more luck (maximum -100) and positive scores indicating more skill (maximum +100). Participants then answered six questions about the opponent on an 11-point Likert scale (1 = Strongly Disagree, 11 = Strongly Agree). Our only a priori hypothesis was that conditions with unexploitable opponents should be rated with more luck and conditions with exploitable opponents should be rated with more skill. We considered the rest of these data exploratory in nature.

Working memory task

A short, modified version of an operation span (OSPAN) task (after [8-9]) was used to assess participants' working memory. The task was obtained from Neurobehavioral Systems online resources. In the task, participants had to solve equations while memorizing a string of letters, with the list length (number of letters to recall) increasing gradually, starting at 2. The letters used were similar to those used by Unsworth, Heitz & Engle (2005) in their automated OSPAN task. Each trial started with an equation such as "5 + (1*2) = 7" presented on screen. Participants had to indicate whether the equation presented was correct or incorrect using the left and right arrow keys, respectively. After the participant had given their response, a letter was immediately presented on screen for 1000ms, after which the next equation was presented; this continued until the list length at the current trial was reached, at which point the participant had to recall the string of letters in the correct order. Participants completed three trials at a list length and proceeded to the next set of three trials at a longer list length, only if they had recalled the string correctly on at least two of the three trials. Participants were instructed to not guess the answers to the equation tasks and to try and be sure they get the answers right. Each participant completed a short training phase with a minimum of two trials at a list length of two; the training trials would repeat until the participant had at least 50% correct recall.

Our a priori predictions were that individuals with higher working memory estimates should show higher win rates during the exploitable conditions since successful performance relied on applying a series of rules contingent on the outcome of the previous trial. Moreover, individuals with lower working memory estimates should more closely approximate MES, since reduced WM capacity predicted better performance when participants were asked to produce random sequences [10].

Executive control task

An arrow flanker task (based on [11]) was used to measure executive control. The task was obtained from Neurobehavioral Systems online resources. Participants used two keys on a keyboard to respond to a central arrow, pressing the left control key with their left index finger for arrows pointing left and the right control key with their right index finger for arrows pointing right. The central arrow (< or >) appeared directly at the location of a central fixation cross that was presented before each trial and was flanked by three arrows both to the left and right. The fixation cross was presented for 200ms, after which the central arrow and flankers on both sides of the central arrow were presented for 200ms. Each flanker was either a right- or left-facing arrow of the same size as the central arrow, approximately 3.4cm x 3.4cm. In congruent trials, each flanker was facing the same direction as the central arrow; in incongruent trials, some of the flankers were facing the opposite direction. After the stimulus presentation, participants had 500ms to respond before the initiation of the next trial. Participants completed a total of 96 trials, divided into two blocks of 48 trials, with an even number of left and right central arrows and congruent and incongruent flankers in both blocks.

Our a priori prediction was that individuals with higher executive control estimates should more closely approximate MES (also after [10]).

Results

*Perception of luck / skill* *and confidence*

We analysed average confidence ratings and the confidence ratings' correlations with win-rates as per Experiment 1 (see Supplementary Table B1). For the average confidence ratings, there was no significant main effect of strategy [F(1, 39) = 1.68, MSE = .52, *p* = .202, ƞ_p_^2^ = .04], no main effect of value [F(1, 39) = 0.94, MSE = .16, *p* = .338, ƞ_p_^2^ = .02] and no two-way interaction [F(1, 39) = 0.75, MSE = .21, p = .387, ƞ_p_^2^ = .02]. For the correlational analyses, eight participants had to be excluded due to no variance in their stated confidence in at least one block. The average correlation coefficients (inverse Fisher transformed) were -.051 *(low value, unexploitable)*, -.001 *(high value, unexploitable),* -.017 *(low value, exploitable)* and -.106 *(high value, exploitable)*. None of the correlations were significantly different from 0 (*p* > .05). There was also no significant main effect of opponent [F(1, 31) = 0.21, MSE = .20, p = .650, ƞ_p_^2^ < .01], or value [F(1, 31) = 0.15, MSE = .09, p = .706, ƞ_p_^2^ < .01] and no interaction [F(1, 31) = 0.89, MSE = .17, p = .353, ƞ_p_^2^ = .03]. We do not have a clear understanding of why the effects on confidence in Experiment 1 did not replicate. One possibility is that asking *both* questions about skill / luck at the end of each block and measuring confidence every 9^th^ trial, in Experiment 2 might have interfered with measurement.

We analysed participants' attribution of condition outcomes to luck or skill using a two-way repeated-measures ANOVA with opponent strategy (*exploitable*, *unexploitable*) and value (*high*, *low*) entered as factors. There was a significant main effect of opponent [F(1, 39) = 28.96, MSE = 2695.82, *p* < .001, *ƞ_p_^2^* = .42] but no main effect of value [F(1, 39) = 0.42, MSE = 1626.51, p = .520, ƞ_p_^2^ = .01] and no interaction [F(1, 39) = 0.10, MSE = 1679.89, p = .757, ƞ_p_^2^ < .01]. Participants indicated they believed more skill than luck drove the results when playing against the *exploitable* opponents (M = 26.65, SE = 7.36) and more luck than skill drove the results than when playing against *unexploitable* opponents (M = -17.52, SE = 6.57).

Questionnaires

Participants' attribution of condition performance to luck or skill were analysed using a two-way repeated measures ANOVA with the factors of opponent (unexploitable, exploitable), value (low, high). There was a significant main effect of opponent [F(1,39) = 28.966, MSE = 2695.828, p < .001, ƞ_p_^2^ = .426]. There was no main effect of value or an interaction effect (both F's < 1). Players reported more luck against unexploitable opponents (M = -17.525, SE = 6.572) and more skill against the exploitable opponents (M = 26.658, SE = 7.368).

These broadly accurate assessments of opponency were also consistent with higher agreement for “my opponent was playing randomly” in the unexploitable condition, and, higher agreement for “the opponent was responding to my moves” in the exploitable condition (see Supplementary Table B1). Also of note were higher agreement for the items “the opponent was responding to my moves” and “my opponent could predict what I was doing” for the unexploitable (random) opponents in Experiment 2. This would appear consistent with the higher ratings of co-presence and agency reported for the unexploitable (random) opponents in Experiment 1 (see Supplementary Table A2).

Working memory task

The effect of working memory on the rate of optimal choices against the exploitable opponent at trial n+1 was analyzed. The span variable used was the last list length where the participant had correct recall on at least two of the three trials for that span length. Four participants were excluded due to an overall accuracy less than 85% on the distractor task in the OSPAN (as per [9]). A further four participants were excluded due to having failed the memory span task on the first trial. For the remaining thirty-two participants (M = 5.063, SE = 0.330), we ran two-way repeated measures ANCOVA with value (high, low) and outcome at trial n (win, lose, draw) entered as factor and working memory span as covariate. There was no significant main effect of the covariate and no significant interactions with the covariate (all F's < 1).

In order to assess the potential contribution of working memory on the ability to express a mixed-equilibrium strategy in the unexploitable conditions, deviation from randomness was calculated by deducing the absolute rate of each move type (stay, upgrade, downgrade) from 33.3%. This was done with respect to all three outcomes (*win*, *lose*, *draw*) and both values (low, high; see Supplementary Table B2). There was no significant main effect of the covariate (F < 1), no significant interaction effects with value or outcome (F's < 1) and no three-way interaction effect [F(2,60) = 1.483, MSE = .003, *p* = .235, ƞ_p_^2^ = .047].

Executive control task

A similar analysis regarding deviation from randomness was conducted on executive control estimates, taken as the difference between median reaction times for the congruent (M = 416, SE = 11) and incongruent (M = 487, SE = 12) stimuli. As a manipulation check for the flanker task, responses to incongruent stimuli were slower than that to congruent stimuli (t[39] = 12.483, *p* < .001). The executive control covariate had no significant main effect [F(1,38) = 2.410, MSE = .010, p = .129, ƞ_p_^2^ = .060], had no significant interaction effects with value [F(1,38) = 1.392, MSE = .005, p = .245, ƞ_p_^2^ = .035] or outcome (F < 1), and there was no three-way interaction between the factors and covariate (F < 1).

| *Supplementary Table B1.* Confidence measures and correlations with win-rate as a function of opponent and value in Experiment 2 (nominal means) | | | |
| --- | --- | --- | --- |
| On-line confidence measure (range: 1 – 5) | | | |
| *Unexploitable opponent* | | *Exploitable opponent* | |
| *Low value* | *High value* | *Low value* | *High value* |
| 2.955 (.069) | 2.830 (.103) | 3.040 (.125) | 3.043 (.126) |
| Confidence measure / win-rate correlations (mean Fisher transformed z values) | | | |
| *Unexploitable opponent* | | *Exploitable opponent* | |
| *Low value* | *High value* | *Low value* | *High value* |
| -.051 (.063) | -.002 (.074) | -.018 (.056) | -.107 (.089) |
| Note: Standard error in parentheses. | | | |

| Supplementary Table B2. Results from the six questions “I think…” presented at the end of each block (range: 1 = Strongly Disagree, 11 = Strongly Agree). | | | | |
| --- | --- | --- | --- | --- |
|  | Unexploitable Opponent | | Exploitable Opponent | |
|  | *Low value* | *High value* | *Low value* | *High value* |
| Q1.“the opponent was responding to my moves” | 6.700 (.421) | 6.500 (.453) | 4.700 (.502) | 5.300 (.541) |
| Q2. “the opponent played according to a pattern” | 5.675 (.441) | 5.125 (.442) | 8.275 (.460) | 8.275 (.439) |
| Q3. “my opponent was somehow cheating” | 3.225 (.415) | 3.350 (.459) | 2.300 (.327) | 2.825 (.402) |
| Q4. “my opponent changed their strategy at some point during the block” | 6.900 (.454) | 5.775 (.454) | 6.350 (.558) | 6.675 (.486) |
| Q5. “my opponent could predict what I was doing” | 6.225 (.448) | 5.600 (.512) | 4.225 (.444) | 4.400 (.445) |
| Q6. “my opponent was playing randomly” | 3.800 (.388) | 3.800 (.410) | 2.825 (.343) | 2.550 (.263) |
| Note: Standard error in parentheses. | | | | |

| Supplementary Table B3. Absolute average deviation from randomness against unexploitable opponents as a function of outcome and value | | |
| --- | --- | --- |
|  | *Low value* | *High value* |
| *Win* | .152 (.013) | .128 (.012) |
| *Lose* | .115 (.010) | .129 (.010) |
| *Draw* | .097 (.010) | .120 (.012) |
| Note: Standard error in parentheses. | | |

References

1. Losecaat Vermeer, A. B., & Sanfey, A. G. (2015). The effect of positive and negative feedback on risk-taking across different contexts. *PLoS ONE*, *10*(9), 1–13. <http://doi.org/10.1371/journal.pone.0139010>

2. Williams, P., Heathcote, A., Nesbitt, K., & Eidels, A. (2016). Post-error recklessness and the hot hand. *Judgement and Decision Making*, *11*, 174–184.

3. Pulford, B. D., Colman, A. M. & Loomes, G. (2018). Incentive magnitude effects in experimental games: Bigger is not necessarily better. *Games*, *9*: 4.

4. Robinson, L. J., Stevens, L. H., Threapleton, C. J. D., Vainiute, J., McAllister-Williams, H., & Gallagher, P. (2012). Effects of intrinsic and extrinsic motivation on attention and memory. *Acta Psychologica, 141(2),* 243-249. <https://doi.org/10.1016/j.actpsy.2012.05.012>.

5. Spaniol, J., Bowen, H. J., Wegier, P., & Grady, C. (2015). Neural responses to monetary incentives in younger and older adults. *Brain Research*, *1612*, 70–82. <http://doi.org/10.1016/j.brainres.2014.09.063>

6. Smith, V. L., & Walker, J. M. (1993). Monetary Rewards and Decision Costs in Experimental Economics. *Economic Inquiry*, *31*(2), 245–61. http://doi.org/10.1111/j.1465-7295.1993.tb00881.x

7. Achtziger, A., Alós-Ferrer, C., Hügelschäfer, S., & Steinhauser, M. (2015). Higher incentives can impair performance: Neural evidence on reinforcement and rationality. *Social Cognitive and Affective Neuroscience*, *10*(11), 1477–1483. <http://doi.org/10.1093/scan/nsv036>

8. Turner, M. L., & Engle, R. W. (1989). Is working memory capacity task dependent? Journal of Memory and Language, 28(2), 127-154. <http://doi.org/10.1016/0749-596X(89)90040-5>

9. Unsworth, N., Heitz, R. P., & Engle, R. W. (2005). An automated version of the operation span task, 37(3), 498-505. <http://doi.org/10.3758/bf03192720>

10. Terhune, D. B., & Brugger, P. (2011). Doing better by getting worse: Posthypnotic amnesia improves random number generation. PLoS ONE, 6(12), 8-11. <http://doi.org/10.1371/journal.pone.0029206>

11. Eriksen, B. A., & Eriksen, C. W. (1974). Effects of noise letters upon the identification of a target letter in a nonsearch task. Perception & Psychophysics, 16(1), 143­149. <http://doi.org/10.3758/BF03203267>
