# Supplementary material for: Breaking the bonds of reinforcement: Effects of trial outcome, rule consistency and rule complexity against exploitable and unexploitable opponents
Source: PLoS One. 2022 Feb 2;17(2):e0262249. doi: 10.1371/journal.pone.0262249 (PMC8809577; doi:10.1371/journal.pone.0262249)
Supplement: S3 File — (DOC) [file pone.0262249.s003.doc]

**ID:** participant identifier. NOTE: Different participants across experiments.

**Opponent:** opponent condition in the experiments: different across experiments

**Outcome_value:** value condition in the experiments: different across experiments

**R_prop:** percentage of Rock decisions out of 90 rounds within the condition

**P_prop:** percentage of Paper decisions out of 90 rounds within the condition

**S_prop:** percentage of Scissors decisions out of 90 rounds within the condition

**R_num:** raw number of Rock decisions out of 90 rounds within the condition

**P_num:** raw number of Paper decisions out of 90 rounds within the condition

**S_num:** raw number of Scissors decisions out of 90 rounds within the condition

**W_prop_total:** percentage of trials won out of 90 rounds within the condition

**L_prop_total:** percentage of trials lost out of 90 rounds within the condition

**D_prop_total:** percentage of trials drawn out of 90 rounds within the condition

**W_num_total:** raw number of trials won out of 90 rounds within the condition

**L_num_total:** raw number of trials lost out of 90 rounds within the condition

**D_num_total:** raw number of trials drawn out of 90 rounds within the condition

**W_num_eligible:** number of trials won excluding the last trial of the condition, thus eligible for analysis of effects of outcome at trial n on choice at trial n+1

**L_num_eligible:** number of trials lost excluding the last trial of the condition, thus eligible for analysis of effects of outcome at trial n on choice at trial n+1

**D_num_eligible:** number of trials drawn excluding the last trial of the condition, thus eligible for analysis of effects of outcome at trial n on choice at trial n+1

**WS_num:** number of win-stay decisions following the eligible win trials

**WU_num:** number of win-upgrade decisions following eligible win trials

**WD_num:** number of win-downgrade decisions following eligible win trials

**LS_num:** number of lose-stay decisions following eligible lose trials

**LU_num:** number of lose-upgrade decisions following eligible lose trials

**LD_num:** number of lose-downgrade decisions following eligible lose trials

**DS_num:** number of draw-stay decisions following eligible draw trials

**DU_num:** number of draw-upgrade decisions following eligible draw trials

**DD_num:** number of draw-downgrade decisions following eligible draw trials

**WS_prop:** percentage of win-stay decisions (raw number of decision type divided by raw number of eligible outcome trials)

**WU_prop:** percentage of win-upgrade decisions (raw number of decision type divided by raw number of eligible outcome trials)

**WD_prop:** percentage of win-downgrade decisions (raw number of decision type divided by raw number of eligible outcome trials)

**LS_prop:** percentage of lose-stay decisions (raw number of decision type divided by raw number of eligible outcome trials)

**LU_prop:** percentage of lose-upgrade decisions (raw number of decision type divided by raw number of eligible outcome trials)

**LD_prop:** percentage of lose-downgrade decisions (raw number of decision type divided by raw number of eligible outcome trials)

**DS_prop:** percentage of draw-stay decisions (raw number of decision type divided by raw number of eligible outcome trials)

**DU_prop:** percentage of draw-upgrade decisions (raw number of decision type divided by raw number of eligible outcome trials)

**DD_prop:** percentage of draw-downgrade decisions (raw number of decision type divided by raw number of eligible outcome trials)

**Mean_confidence:** mean of the reverse coded confidence measure (taken every 9^th^ trial of each block), range: 1-5, with 5 indicating high confidence of win and 1 indicating high confidence of loss

**Confidence_win_r:** correlation between the win-rate from each 9-trial section and the confidence reported on the 9^th^ trial (note: for convenience, the win-rate calculation includes the 9^th^ trial, though the outcome of the 9^th^ trial is only apparent to the participant after giving the confidence rating). NA indicates correlation could not be calculated due to no variance in confidence ratings.

**Confidence_win_z:** fisher-transformed correlation between the win-rate from each 9-trial section and the confidence reported on the 9^th^ trial

Experiment 1 -specific measures:

**GEQ01-GEQ16:** questions in the Game Engagement Questionnaire in Experiment 1. NA indicate missing responses (missing participant was excluded from all questionnaire analyses in Supplementary Materials A)

**Copres1-Copres7:** questions in the Co-presence Questionnaire in Experiment 1. NA indicate missing responses (missing participant was excluded from all questionnaire analyses in Supplementary Materials A)

**ANT01-ANT08:** questions in the Anthropomorphism Questionnaire in Experiment 1. NA indicate missing responses (missing participant was excluded from all questionnaire analyses in Supplementary Materials A)

**GEQ_mean:** mean of GEQ items in Experiment 1. NA indicate missing responses (missing participant was excluded from all questionnaire analyses in Supplementary Materials A)

**CoPres_mean:** mean of Co-presence items in Experiment 1. NA indicate missing responses (missing participant was excluded from all questionnaire analyses in Supplementary Materials A)

**ANT_mean:** mean of the five anthropomorphic state items (ANT01-ANT05) in the Anthropomorphism questionnaire. NA indicate missing responses (missing participant was excluded from all questionnaire analyses in Supplementary Materials A)

**NonANT_mean:** mean of the three non-anthropomorphic state items (ANT06-ANT08) in the Anthropomorphism questionnaire. NA indicate missing responses (missing participant was excluded from all questionnaire analyses in Supplementary Materials A)

**Honesty:** mean of the honesty/humility items of the 60-item HEXACO questionnaire. NA indicate missing responses (missing participant was excluded from all questionnaire analyses in Supplementary Materials A)

**Emotionality:** mean of the emotionality items of the 60-item HEXACO questionnaire. NA indicate missing responses (missing participant was excluded from all questionnaire analyses in Supplementary Materials A)

**Extraversion:** mean of the extraversion items of the 60-item HEXACO questionnaire. NA indicate missing responses (missing participant was excluded from all questionnaire analyses in Supplementary Materials A)

**Agreeableness:** mean of the agreeableness items of the 60-item HEXACO questionnaire. NA indicate missing responses (missing participant was excluded from all questionnaire analyses in Supplementary Materials A)

**Conscientousness:** mean of the conscientiousness items of the 60-item HEXACO questionnaire. NA indicate missing responses (missing participant was excluded from all questionnaire analyses in Supplementary Materials A)

**Openness:** mean of the openness items of the 60-item HEXACO questionnaire. NA indicate missing responses (missing participant was excluded from all questionnaire analyses in Supplementary Materials A)

Experiment 2 -specific measures:

**Luck_skill_slider:** luck/skill measure used in Experiment 2, range: -100 (pure luck) to 100 (pure skill)

**Q01-Q06:** items in the post-block questionnaire (see Supplementary Materials B for items)

**OSPAN:** highest successful working memory span in the OSPAN task (NA indicates participant excluded due to failed task)

**EXCON_CONG:** median RT in congruent trials in the Flanker task

**EXCON_INCONG:** median RT in incongruent trials in the Flanker task

**EXCON_DIFF:** EXCON_INCONG – EXCON_CONG: difference between incongruent and congruent trial Rts
